# Long-Term Effects of Chronic Light Pollution on Seasonal Functions of European Blackbirds (*Turdus merula*)

**DOI:** 10.1371/journal.pone.0085069

**Published:** 2013-12-20

**Authors:** Davide M. Dominoni, Michael Quetting, Jesko Partecke

**Affiliations:** 1 Department of Migration and Immuno-ecology, Max Planck Institute for Ornithology, Radolfzell, Germany; 2 Department of Biology, University of Konstanz, Konstanz, Germany; 3 Institute of Biodiversity, Animal Health and Comparative Medicine, University of Glasgow, Glasgow, United Kingdom; CNRS, France

## Abstract

Light pollution is known to affect important biological functions of wild animals, including daily and annual cycles. However, knowledge about long-term effects of chronic exposure to artificial light at night is still very limited. Here we present data on reproductive physiology, molt and locomotor activity during two-year cycles of European blackbirds (Turdus merula) exposed to either dark nights or 0.3 lux at night. As expected, control birds kept under dark nights exhibited two regular testicular and testosterone cycles during the two-year experiment. Control urban birds developed testes faster than their control rural conspecifics. Conversely, while in the first year blackbirds exposed to light at night showed a normal but earlier gonadal cycle compared to control birds, during the second year the reproductive system did not develop at all: both testicular size and testosterone concentration were at baseline levels in all birds. In addition, molt sequence in light-treated birds was more irregular than in control birds in both years. Analysis of locomotor activity showed that birds were still synchronized to the underlying light-dark cycle. We suggest that the lack of reproductive activity and irregular molt progression were possibly the results of i) birds being stuck in a photorefractory state and/or ii) chronic stress. Our data show that chronic low intensities of light at night can dramatically affect the reproductive system. Future studies are needed in order to investigate if and how urban animals avoid such negative impact and to elucidate the physiological mechanisms behind these profound long-term effects of artificial light at night. Finally we call for collaboration between scientists and policy makers to limit the impact of light pollution on animals and ecosystems.

## Introduction

The study of the ecological consequences of artificial light at night has received great interest in the last decade, particularly in the context of the effects on wildlife [1]. Light at night has been shown to affect the composition of invertebrate communities [2], the foraging behavior of beach mice [3] and shorebirds [4], the stress response of tuna [5] and the commuting strategies of bats [6]. In songbirds, artificial lighting has long been thought to affect daily and seasonal cycles [7], and recent studies have provided correlational demonstration of such effects [8–10]. 

In a recent study, we showed that European blackbirds (*Turdus merula*) exposed to a light intensity at night of 0.3 lux, representative of the intensity measured with light loggers on individual blackbirds in an urban area, developed the reproductive system almost a month in advance, and also moulted earlier, than conspecifics exposed to dark nights [10]. Furthermore, during the testicular regression phase blackbirds originating from urban areas responded differently than blackbirds from the forest when exposed to the light at night treatment, in that urban birds ended the reproductive cycle sooner than rural birds. These results already indicated pronounced effects of low light intensities at night on the timing of reproductive physiology. However, our knowledge about long-lasting effects of such low light intensities at night on the seasonal organization of urban living animals is still limited. In this context it is important to consider how photoperiodic information is integrated in avian species living in temperate areas. In general, these bird species enter a state of photorefractoriness after the breeding season [11]. That is, long days in summer are no longer photo-stimulatory, and gonads start to regress. In order to re-grow their reproductive system birds need to become photosensitive again, and this is accomplished by exposure to short days (e.g. in autumn). Once photosensitivity is acquired, the increase in day length (e.g. during winter or spring) induces the development of the gonads. The seasonal alternation of the two phases, photorefractoriness and photosensitivity, ensures the maintenance of functional reproductive cycles [12]. If this alternation is broken or disrupted for example by artificial changes in daylength, birds may get stuck in one phase [13]. 

In urban areas, it could be possible that birds interpret low light intensities at night as a constant long day, which would prevent them from breaking phototorefractoriness in late autumn [13] and from reproducing during the following spring. Alternatively, light at night may not perturb the biological significance of seasonal change in photoperiod: That is, short days in autumn would allow birds to recover photosensitivity and the increasing daylength in early spring would stimulate reproductive growth. In this case we would expect the same differences in reproductive timing found during the first annual cycle, that is, birds exposed to light at night should start testicular growth earlier than birds under dark nights. We tested this hypothesis by monitoring changes in testicular size, plasma testosterone concentration and molt over two consecutive annual cycles. Since the data from the first year has been already presented elsewhere [10], we focus here on the second annual cycle.

## Methods

### Ethics Statement

The European blackbird is a common and widespread songbird in Europe, and it is listed as species of “Least Concern” in the IUCN Red List. Bird-catching was conducted with mist-nets at dawn and was permitted for both locations (Raisting forest and City of Munich) by the Max Planck Institute for Ornithology and Regierung von Oberbayern, Munich, Germany (permit number: 55.1-8642.3-17-2008). All the experimental procedures in the laboratory were approved by the Max Planck Institute for Ornithology and the Department 35 of the Regional Commission Freiburg, Baden Württemberg, Germany. Blood samples were collected by puncturing the brachial vein. Measurements of testicular size (see below for details) were conducted by treating birds with Isoflurane anaesthesia. No bird died or was harmed by this procedure.

### Animals and experimental set-up

Detailed explanations of our experimental set-up can be found in our recent paper [10]. Briefly, in summer 2010 we caught wild rural (N = 20) and urban (N = 20) male European blackbirds from our study populations (rural population: forest close to the village of Raisting and urban population: City of Munich). Birds were transported to our facilities in Radolfzell, Germany and, after few months in outdoor aviaries, they were divided into two groups and placed in indoor cages in two separated rooms. Each room contained an equal number of rural and urban individuals ((N rural = 10, N urban = 10). All birds were initially exposed to natural local photoperiod. Day-time light was provided by dimmable fluorescent tube lights (Biolux 36 W, Osram, Germany) emitting light at wavelengths covering the human visible spectrum. Day-time light intensity in each cage ranged between 250 and 1250 lux. Night-time light was provided by dimmable incandescent lamps (SLV Elektronic, Germany, wavelength range ~ 450-950 nm) and intensity was ~ 0.0001 lux. On Dec. 18^th^, 2010, one of the two groups (from now on called “experimental”) was subjected to light at night of 0.3 lux, while the other group (hereafter called “control”) stayed under 0.0001 lux at night. The light intensities at night in both rooms were calibrated on data obtained from light-loggers deployed on free-roaming urban and rural blackbirds, as previously shown [10]. In addition, to verify the validity of our treatment, we placed light loggers on 39 birds and record light levels for one full night. The light intensity in the experimental room, calculated following [10], ranged between 0.12 and 0.35 lux. The experiment lasted until August 31^st^, 2012. One urban bird in the control group died on April 1^st^, 2011. Birds could hear but not see each other. Food (Granvit, Chemi-Vit, Italy) and drinking water were available ad libitum. 

### Testicular measurement and hormone analysis

We collected blood samples by puncturing the brachial vein from every individual on December 8^th^, 2010, and thereafter every month between January-July 2011 and November 2011-July 2012. Blood was immediately centrifuged and plasma separated from red blood cells and stored at – 80 °C. Plasma samples were analyzed for testosterone concentration (T) in July 2012 via a commercial enzyme immunoassay (EIA) kit (# 901-065, Enzo Life Sciences, NY, USA). Plasma samples from each individual for two reproductive cycles (18 samples per bird) were analyzed on the same plate. Samples from two individuals were included on each plate. A total of 20 assays were run. Detection limit was 5.67 pg/tube plasma T. The mean intra-assay coefficient of variation of two replicate standards per plate was 6.9 % and the inter-assay coefficient of variation was 11.9 %. Further details about sampling techniques, extraction method and analysis can be found in [10].

We measured the size of testes by laparotomies [14], one week after each blood sampling session, starting December 15^th^, 2010. Testicular size was assessed through laparotomy [14]. Incisions were made under Isoflurane anesthesia (CP-Pharma, Germany). The width of the left testis was measured to the nearest 0.1 mm. Incisions were treated with Actihaemyl gel (Meda Pharma GmbH, Germany) and sealed with Histoacryl (Braun, Germany). All birds recovered rapidly from the procedure.

### Assessment of body mass and molt

We assessed body mass and fat score of birds on the day they were moved indoor, and thereafter every month. Birds were weight with a laboratory balance (KERN PCB 1000-2, precision 0.1 g, KERN, Germany) and the amount of subcutaneous fat was scored on a 0-8 scale following [15]. In 2011, we recorded the state of flight feather molt on a weekly basis starting on March 2011, using a method modified from [16]. Briefly, we scored the molt status of the first 10 primaries and the first 6 secondaries, on a 0-5 scale, where 0 = no molt and 5 = completed molt. We then summarized all scores for all feathers for each individual at each molt check. Six experimental birds (rural, N = 3, urban, N = 3) did not finish molt, but they were anyway included in the analysis and the time of molt end was defined as the date when they did not grow feathers anymore. In 2012 we could not check molt every month. We therefore measured molt status only once, on August 13^th^, 2012. 

### Locomotor activity

Locomotor activity was recorded continuously over the entire duration of the experiment through a passive infrared sensor mounted on each cage (Intellisense, CK Systems, Eindhoven, The Netherlands). Movements were counted and stored as two-minute bins into a computer (min = 0, max = 99). 

We used the activity data to test whether experimental birds were synchronized to the simulated photoperiod of the 24 h cycle or whether they interpreted the light treatment as a 24 h long day. To this end we quantified i) the length of their activity period and ii) to what extent their daily activity was still synchronized to the onset and end of the day, i.e. the morning and evening twilights. We selected the activity data in late autumn/early winter, because this is the time of the year when birds living at temperate latitudes seem to recover sensitivity to light after months of photorefractoriness [12,17]. We used the period between November 1^st^ and December 27^th^, 2011, and pooled data on a weekly basis, for each bird. Data were imported in the ImageJ plugin ActogramJ [18], which allows the identification of the main periodicity of activity cycles and calculation of the average activity at each time point of a day (in our case two min. bins). The main periodicity for each bird in each week was estimated through a Lomb-Scargle periodogram [19]. The onset of daily activity was estimated as the time when the average activity between two hours before and two hours after morning twilight crossed a threshold value of 20 bouts per bin (maximum value is 99). We used the same procedure for the evening twilight/end of daily activity. The average activity during the selected hours was 10.9 in the morning and 7.5 in the evening. Therefore a threshold value of 20 likely reduces the chances to detect a change in activity status when it is not present, and is thus a conservative approach to the estimation of the time of activity onset and end. 

### Statistical analysis

Statistical analyses were conducted with software R 2.15.0. All tests were two-tailed and we applied a significance level α = 0.05. When mixed models were used, individuals were always included as random intercepts to account for non-independency of repeated measures. In linear mixed models (LMMs) we first assessed which was the best model by comparing AIC values, and then we evaluated the significance of model parameters using a Monte Carlo Markov Chain (MCMC) approach through the function *pvals.fnc* in the R package *languageR* [20]. P-values (pMCMC) were calculated based on the posterior distribution of model parameters (50000 iterations). In all other models P-values were computed from the t-distribution. When a significant interaction was present in a LMM, our inference was based on multiple comparisons of 95 % confidence intervals (CI) of the estimates for each level of the interaction. CI were calculated using the function *sim* in the R package arm [21]. We considered the means of two groups to be significantly different if the CI of the estimate for one group did not include the means of the other groups. 

Variation in testicular size and testosterone concentration over the two reproductive season was analyzed by univariate linear mixed-effect models (R package *lme4* [22]). Testicular width or testosterone concentration were included as response variables, log-transformed to reach normality in the distribution of the residuals and homogeneity of variance. We included year, date, second polynomial (quadratic) date, treatment, origin and all 4-ways interactions between treatment, origin, year and either date or 2^nd^ polynomial date as fixed effects. We sequentially removed non-significant interactions. 

In addition, to compare timing of gonadal growth, we used threshold values of testicular width to estimate the date at which birds reached a functional testes width of 5 mm, a threshold that was selected on the assumption that testes start producing sperm at half-maximum volume [23]. The exact date at which testicular growth passed the threshold value was extrapolated for each individual from a four-parameter logistic equation (GraphPad Software, USA). The equation used was: Testicular Size = B + (A - B) / 1 + exp ((C – date) / D), where A = lower asymptote of the curve, B = upper asymptote of the curve, C = response half way between bottom and top, and D = slope of the curve at half way between bottom and top. We tested differences between groups using ANOVAs.

We used LMMs to analyze the variation in body mass over the two years. Body mass and fat scores were included as response variables. We first modelled treatment, origin, date, year and the 4-way interaction as fixed factors. The best model for weight included two-ways interactions between treatment and either date or year, and all main effects. The best model for fat scores included the interaction between treatment and year and all main factors. 

In order to test whether the seasonal timing and pattern of molt was different between treatment groups and populations in 2011, we first analyzed the variation in molt scores of all birds using a general additive mixed model (GAMM, R package *mgcv*, [24]). Treatment, origin, and interaction between treatment and origin were included as parametric terms. The four possible interactions between factors’ levels and date were modelled as smoothed terms. In addition, we analyzed the difference in the time of molt start and end between treatment groups and populations by using univariate generalized linear models (GLMs) with a Poisson error structure and a log-link. The date of molt onset or end was included as response variable, and treatment, origin and their interaction were modelled as fixed factors. To test whether the birds differed in the duration of molt we used a linear model (LM) with the number of days between molt start and end as response variable. Treatment, origin and their interaction were modelled as fixed factors. To analyze the molt data in 2012 we used a LM with molt score as dependent variable, and treatment, origin and feather (primaries: 1-10, secondaries 1-6) as fixed factors. In addition, we tested for normality within each treatment group using Shapiro-Wilk test and then tested for differences between the normal distributions associated with each group using Kolmogorov-Smirnov test. Finally, we analyzed the variation in period length of daily activity in November and December 2011 using LMMs. Period length was included as response variable, while date, treatment, origin and their interactions were included as fixed factors. We used the same models to analyze the variation in onset and end of daily activity during the two months. The time of onset and end of activity was corrected for the average twilight of the week over which we averaged the activity data (see paragraph “Locomotor activity” above). Date, treatment, origin and their interactions were included as fixed factors

## Results

**Figure 1:**
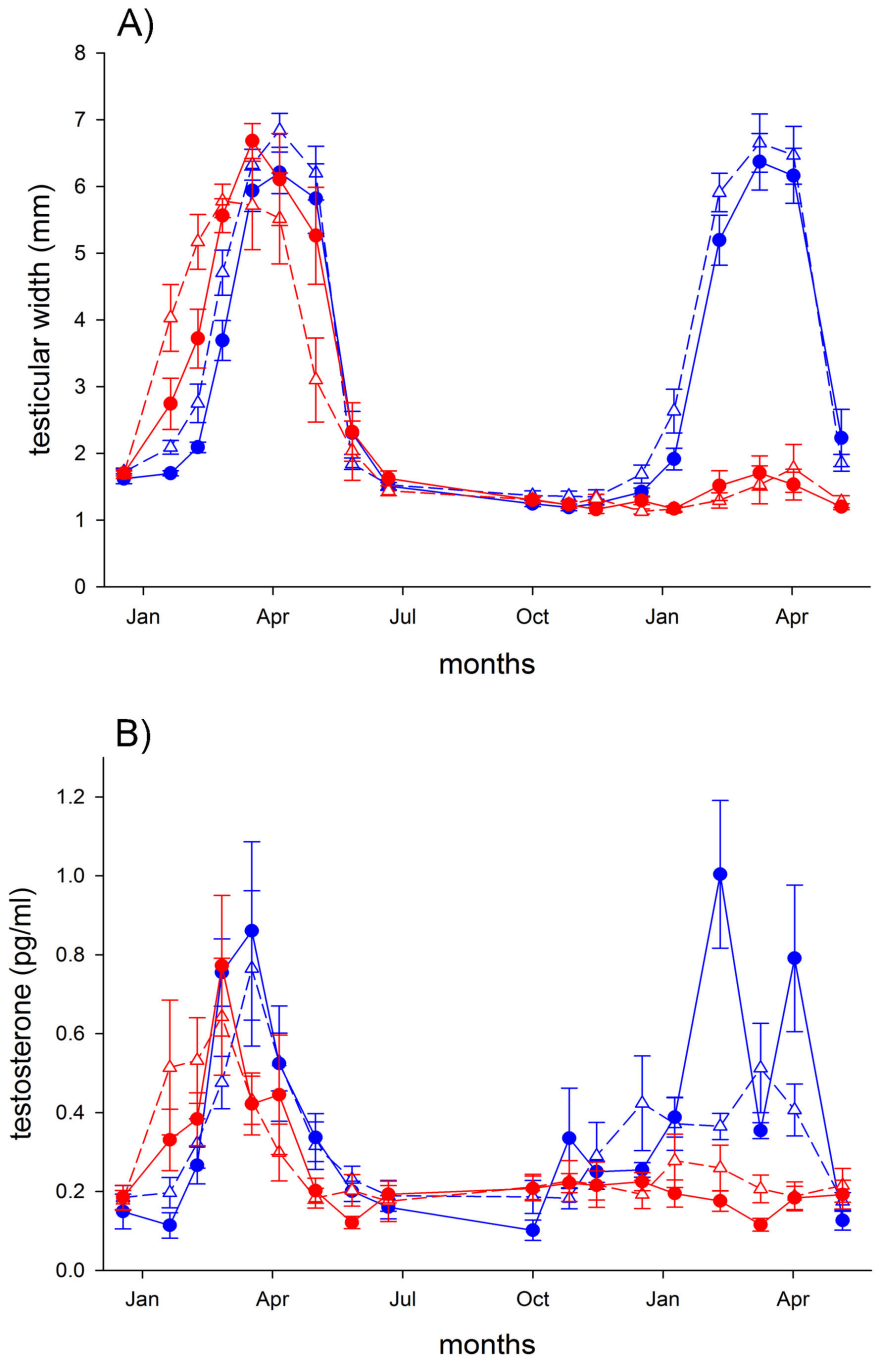
Effect of light at night on seasonal variation in testicular width (A) and plasma testosterone levels (B) in captive adult male European blackbirds (*Turdus merula*). Urban (triangles, dashed lines) and rural (circles, solid lines) blackbirds were exposed to simulated natural photoperiods but with different light intensities at night. Control birds (blue) experienced nights with light intensity of 0.0001 lux, while experimental birds (red) were exposed to constant light of 0.3 lux at night. Birds were measured from December 2010 to June 2012. Data represent mean ± SEM. Sample sizes: control = 20 (10 rural and 10 urban), experimental = 20 (10 rural and 10 urban). One urban bird in the control group died on April 1^st^, 2011.

The analysis of testicular cycles revealed a significant three-way interaction between year, treatment and date (LMM, pMCMC = 0.004, Table 1). While in the first experimental year both treatment groups went through a regular testicular cycle, with experimental birds that were earlier than control birds, in the second experimental year testicular size in the birds exposed to light at night remained low and comparable to baseline levels for the entire reproductive cycle (Figure 1). Conversely, birds exposed to dark nights showed a regular gonadal cycle also in the second experimental year. In addition, in this treatment group urban birds showed earlier testicular growth than rural conspecifics, as previously shown for the first experimental year [10]. Specifically, urban birds reached the threshold of 5 mm in testicular width 9 days before rural individuals (calendar dates ± SEM: urban = 64.6 ± 3.4, rural = 73.3 ± 2.4), an effect size comparable to that of the first experimental year (8 days, [10]). However this difference was only marginally significant (t-test, t = 2.1, df = 13.02, P = 0.059).

**Table 1:** Variation of testicular size (A) and testosterone levels (B) over two reproductive cycles.

**A) testicular width**				
**parameter**	**estimate**	**SEM**	**t value**	**pMCMC**
intercept	0.73	0.06	11.35	< 0.001
date	0.01	< 0.01	13.63	< 0.001
2^nd^ polynomial date	< -0.01	< 0.01	-15.77	< 0.001
year	-12.38	0.65	-19.06	< 0.001
origin	0.10	0.05	2.11	0.061
treatment	0.39	0.09	4.46	< 0.001
treatment * origin	-0.13	0.07	-1.86	0.091
date * treatment	< 0.01	< 0.01	-3.72	< 0.001
year * treatment	1.35	0.28	4.90	< 0.001
year * date	0.04	< 0.01	17.05	< 0.001
year * date * treatment	< -0.01	< 0.01	-2.79	0.004
**B) testosterone concentration**				
**parameter**	**estimate**	**SEM**	**t value**	**pMCMC**
Intercept	0.29	0.03	8.93	< 0.001
Date	< 0.01	< 0.01	0.87	0.375
2^nd^ polynomial date	< 0.01	< 0.01	0.90	0.371
Year	-0.11	0.05	-2.09	0.034
Origin	< 0.01	0.03	0.19	0.832
Treatment	0.03	0.04	0.78	0.355
date * treatment	< -0.01	< 0.01	-3.47	< 0.001
year * treatment	0.15	0.07	2.00	0.050

Significance of parameters was estimated via Markov Chain Monte Carlo (pMCMC, 50000 iterations)[20].

Models are LMMs (linear mixed models) with log-transformed testicular width or testosterone concentration as response variables.

The best full model for the testosterone concentration during the first and second year revealed significant two-way interactions between treatment and date (LMM, pMCMC < 0.001, Table 1B) and treatment and year (LMM, pMCMC = 0.05, Table 1B). These results mirrored the pattern of the testicular cycle in both years. In the first experimental year plasma testosterone concentration increased earlier in birds exposed to light at night than in the treatment group exposed to dark nights. In the second experimental year, as evident from Figure 1B, plasma T levels in the experimental group remained low for the entire reproductive period, while in control birds T showed normal seasonal variation.

Body mass and fat scores varied over the course of the two years. In particular, body mass and fat scores were lower in the experimental group during the second year, as indicated by the significant interactions between treatment and year for both body mass (LMM, estimate = -4.4, pMCMC = 0.004) and fat scores (LMM, estimate = -0.6, pMCMC < 0.001). 

**Figure 2:**
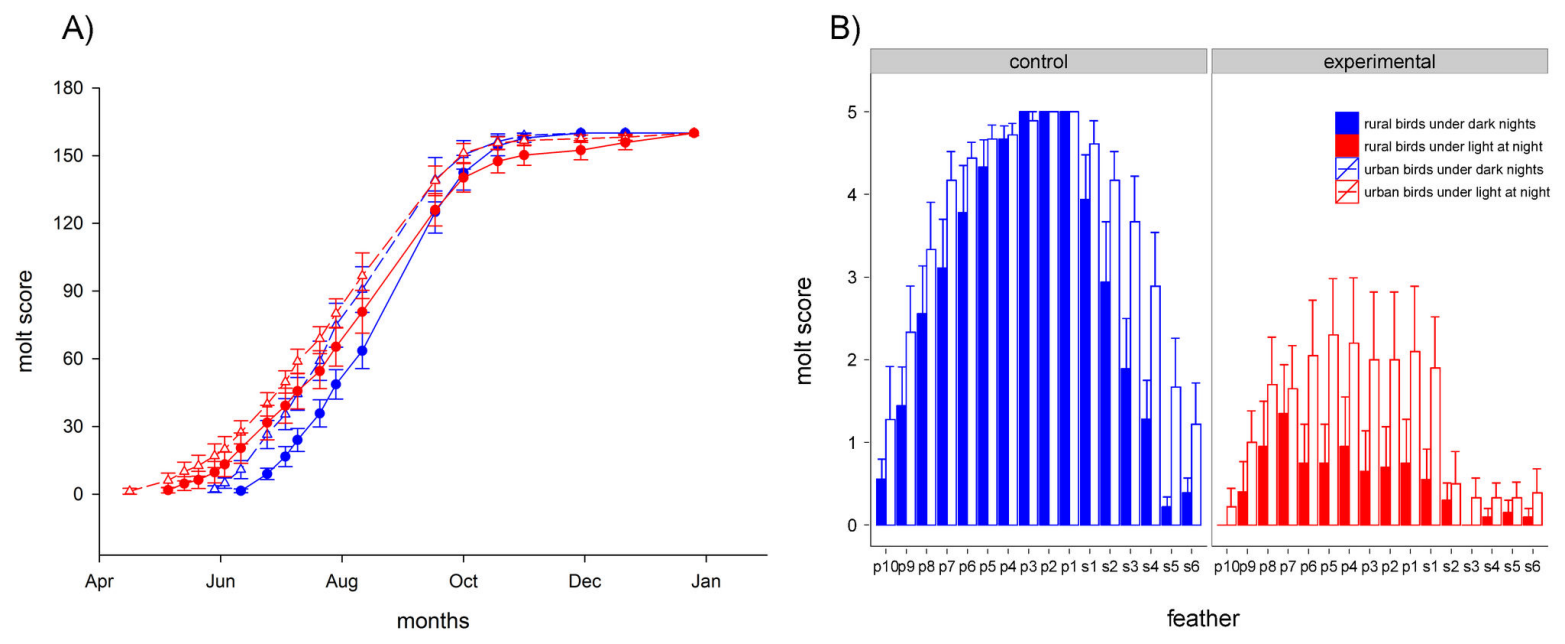
Effect of light at night on molt pattern. We scored the molt condition (0 = no molt, 5 = completed molt) for the ten primary and the first six secondary flight feathers. In the first experimental year (A), molt was measured between April and December (x-axis). Control birds (blue) experienced dark nights, while experimental birds (red) were exposed to constant light of 0.3 lux at night. Triangles and dashed lines depict urban birds, circles and solid lines depict rural birds. Each symbol represents the sum of molt scores for all feathers of each individual, averaged over all individuals of one group. Error bars represent SEM. Six experimental birds, three rural and three urban, did not finish to molt. In the second experimental year (B), we checked molt only once, on August 13^th^. Vertical bars represent the molt score for each feather, averaged over all individuals of one group. Blue bars (left) depict control birds, red bars (right) depict experimental birds. Within each treatment group, blank bars represent urban birds, filled bars represent rural birds. Error bars represent SEM. For details of experimental set-up see Methods and Figure 1.

In the first experimental year the timing and pattern of molt were significantly different between birds exposed to either light at night or dark nights (GAMM, P = 0.042, Figure 2A). Birds under light at night took 37 days more to complete molt (LM, F_2,36_ = 4.29, P = 0.006) than birds under dark nights. This difference was mainly due to an earlier onset of molt in the experimental group, as already reported earlier [10]. The end of molt did not differ between treatment groups (GLM, df = 36, 38, P = 0.158). However, irrespective of the light treatment, timing of molt differed between urban and rural birds: urban birds started and ended to molt earlier than rural conspecifics (molt start: GAMM, P = 0.015; molt end: GLM, df = 36, 38, P = 0.003; Figure 2A). In the second experimental year, we detected significant main effects of treatment, origin and feather number (LM, F_3,599_ = 103.3, R^2^ = 0.35). Birds exposed to light at night were in a disrupted molt state, as indicated by lower molt scores, than birds exposed to dark nights (P < 0.001, mean ± s.e.m.: control = 3.25 ± 0.28, experimental = 0.92 ± 0.13, Figure 2B). As in the first experimental year, urban birds were in a more advanced molt state than rural conspecifics, irrespective of the light treatment (P < 0.001, mean ± s.e.m.: rural = 1.70 ± 0.30, urban = 2.47 ± 0.28, Figure 2B). In birds exposed to dark nights molt normally progressed from the inner primaries and secondaries towards the outer feathers. In the birds exposed to light at night, however, the peak of the molt distribution was skewed towards the 8^th^ and 9^th^ primary (Figure 2B). Indeed, Shapiro-Wilk normality tests indicated that control birds had a higher W-value than experimental birds (control = 0.78, experimental = 0.61), and the Kolmogorov-Smirnov test confirmed that the distributions of molt scores in the two treatment groups were significantly different from each other (D = 0.52, P < 0.001). 

**Figure 3:**
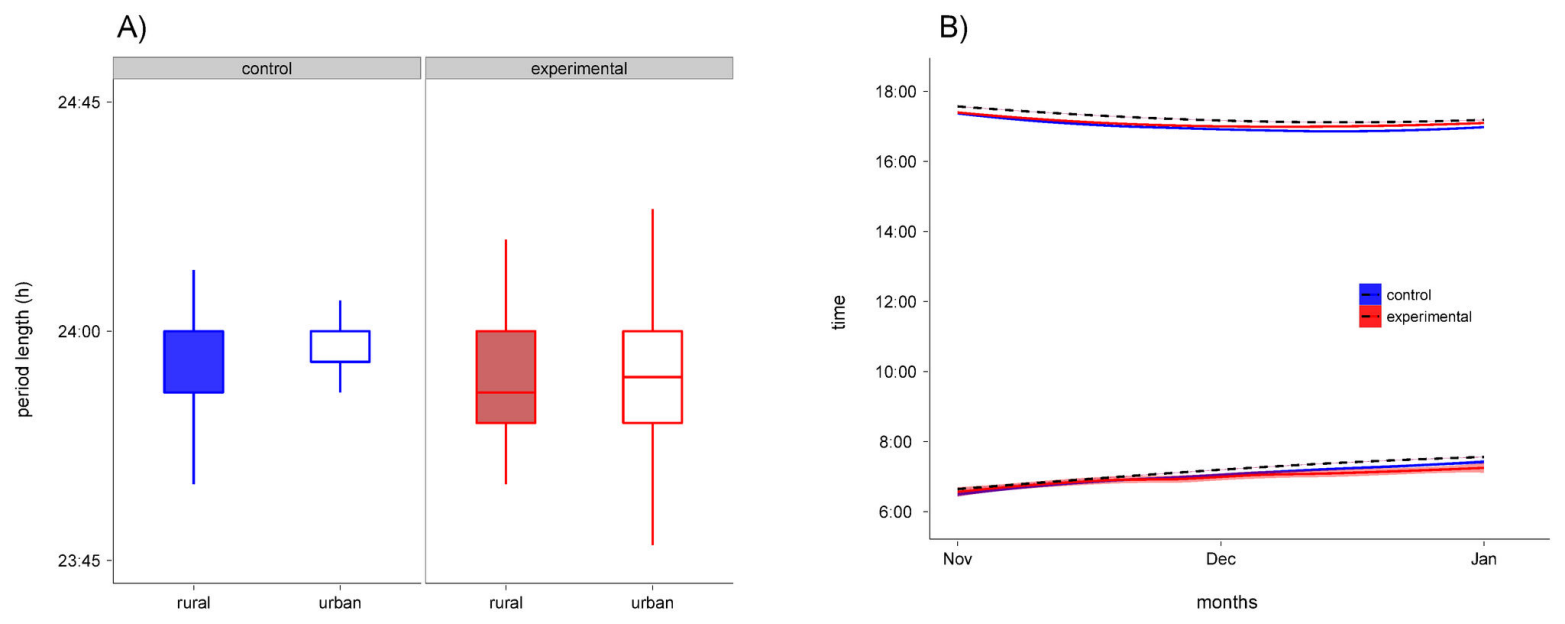
Effect of light at night on period of rhythmicity and entrainment to light/dark cycles. A) We measured the length in hours of the main periodicity of locomotor activity between November 1^st^ and December 27^th^, 2011, hence before the second experimental year, using a Lomb-Scargle periodogram. Average period length was 1437 min and no significant difference was found between either treatment groups (control = blue/left, experimental = red/right) or populations (rural = filled, urban = blank). Box plots represent, from bottom to top: one standard deviation (s.d.) below the mean, lower quartile, median, upper quartile and one s.d. above the mean. B) Onset and end of daily locomotor activity time measured during the same time period of data shown in panel A. We only show data for treatment groups as this facilitates visualization and interpretation of results. Lines and shaded areas (blue = control, red = experimental) represent mean ± SEM. Dashed black lines represent onset of morning twilight and end of evening twilight. For details of experimental set-up see Methods and Figure 1.

The average period length of the daily activity in November and December 2011 was very close to 24 h (1437.2 min, Figure 3A), and did not differ between treatments (LMM, pMCMC = 0.330), populations (LMM, pMCMC = 0.500) or date (LMM, pMCMC = 0.410). The difference between time of onset of activity and onset of morning twilight did not differ between treatment groups during the same months (treatment*date interaction, LMM, pMCMC = 0.160, Figure 3B). Similarly, the difference between the time of end of activity and the end of evening twilight was not different between control and experimental birds (treatment*date interaction, LMM, pMCMC = 0.120, Figure 3B). 

## Discussion

Our study shows that long-term, chronic exposure to very low light intensities at night, which are omnipresent in urban areas, can disrupt important seasonal functions of birds, such as reproduction and molt. During the first monitored reproductive season, European blackbirds exposed to 0.3 lux at night develop reproductive functions, as measured by testicular development and testosterone production, almost a month earlier than conspecifics exposed to dark nights. Irrespectively of the light treatment, urban birds developed functional testes earlier than rural birds. In addition, light-treated birds molted earlier than the control cohort. These results have been previously elsewhere discussed in greater details [10]. 

The most prominent effect, however, occurred in the second reproductive cycle during the second experimental year. Blackbirds exposed to light at night showed no sign of reproductive activity. Both testicular size and testosterone concentration in the blood remained at baseline levels for the entire reproductive season. Conversely, control birds went through a regular cycle which followed the pattern of the first year, i.e. urban birds developed testes earlier than rural individuals, although there was only a tendency for a significant origin effect (Table 1). Why did birds exposed to light at night fail to develop gonads during the second year? A possible scenario is that birds were stuck in a photorefractory state. For instance, European starlings (*Sturnus vulgaris*) which were exposed to constant long days (LD 13:11), grew their gonads in the first year but not in the following seasons [13]. This was interpreted as a failure of these birds to break photorefractoriness under constant long days [13,25]. This observation could also explain the unresponsiveness of the reproductive axis of birds exposed to even low light intensities during night during the second year in our study. The analysis of locomotor activity before the second reproductive year suggests that all birds from the control and treated group were entrained to the underlying light/dark cycle, indicated by synchronization of their onset and end of activities to the civil twilight phases on each day. Thus, they possibly did not interpret the light at night treatment as a longer day (Figure 3). However, activity pattern might be differently regulated than the reproductive system. Indeed, avian photoperiodic time measurement depends on photoreceptors located in the hypothalamus [26] and that regulate a circadian rhythm of photosensitivity to light [27] independently of other components of the circadian system [12], such as locomotor activity. We therefore speculate that, although birds under light at night showed the same daily cycles than the control birds under dark nights, they may have internally interpreted always a longer day due to continuous low light at night. In this context, we have recently shown elsewhere that this light at night treatment is able to reduce melatonin release at night [28]. To what extent the reduction in melatonin release is translated into the detection of a longer day is still unclear. Further experiments aim at measuring expression of specific genes involved in the regulation of daily rhythms and photoperiodic time measurements are probably needed in order to test this hypothesis.

An alternative, non-mutually exclusive hypothesis is that birds exposed to long-term artificial light at night were in a state of chronic stress. We did not collect corticosterone data to confirm this hypothesis, but the fact that experimental birds had reduced body weight and fat scores during the second year possibly may indicate that they could have been chronically stressed. Stress is known to have many effects on vertebrate physiology and behaviour. In particular, stress has been suggested to have negative impacts at various levels of the hypothalamic-pituitary-gonadal axis (HPG), such as down-regulation of gonadotropin-releasing hormone (GnRH) [29], up-regulation of gonadotropin-inhibitory hormone (GnIH/RFRP) and suppression of luteinizing hormone [30], suppression of sexual behavior [31] and ultimately impairment of reproductive activities [32]. In light of these negative effects of chronic stress on reproduction, it may be possible that the lack of reproductive function in the blackbirds exposed to light at night during the second year of the experiment was a result of a chronically stressful situation. However more detailed information on the response of the stress axis is obviously crucial for the understanding of the relationship between light at night, stress and reproduction. 

The light at night treatment also had profound effects on the timing and pattern of molt. The overall duration of the molting period after the first reproductive cycle was longer in experimental than in control individuals. Therefore the rate of molt in the experimental cohort was considerably slower than in the control group. Furthermore, six of the birds exposed to light at night, equally divided between populations, did not complete molt. The mechanism behind the long or uncompleted molt in the experimental group could again be explained by the exposure to very long photoperiods. Decreasing daylengths are known to reduce the duration of molt [33]. We suggest that birds interpreted the light at night treatment as a constant long day and therefore were not exposed to the decreasing daylength necessary to complete molt in a normal time. An alternative explanation could be that the pure calendar effect caused a longer molting period in the experimental groups. Indeed, birds are known to speed up their rate of moult if the start of moult is delayed [33,34]. Thus, experimental birds might have undergone a longer moult simply because they started to moult earlier. During the second year, birds exposed to light at night showed disrupted and irregular molt compared to birds under dark nights. The basic sequence of flight feathers molt is uniform between European passerine species [35]. The inner primaries usually molt earlier than the outer ones, and the same sequence is seen in the secondaries [35]. Conversely, during the second molt cycle in our study, experimental birds molted the outer primaries earlier than the inner ones (Figure 2B). Overall we conclude that light at night can initially advance the timing of onset of molt because birds may experience a long daylength, but on a long-term it can produce an irregular and incomplete sequence of feather replacement. 

The light intensity we used in our experiment was calibrated from data obtained with light loggers deployed on individual free-roaming blackbirds [10]. Although we aimed to simulate the light environment of urban areas as close as possible, a chronic exposure to light at night is probably unrealistic in animals thriving in urban areas. To our knowledge, there are no reports of urban songbirds that failed to show either activation of the reproductive system during the breeding season or irregular molt, but no systematic study has been conducted to exclude this possibility (but see 23). Our experimental light intensity at night (0.3 lux) falls within the range of what free-living blackbirds experience in urban areas during night, and it is well below the average light intensity that we measured underneath common streetlamps in our urban field site (~ 6 lux [10,36]). Furthermore it is also below light intensities used in previous captive studies that investigated the physiological effects of light pollution in different mammal and bird species (from 3.2 to 20 lux [37–39]). Nevertheless, our experimental treatment differed substantially from a natural situation in that birds in cities are more likely to be exposed to variable light intensities at night, both within- and between nights, and not to a chronic and constant administration of artificial light at night. It might be possible that urban birds get exposed to short light pulses during the night and/or perceive the combination of natural daylength and light as night as a subjective longer photoperiod during early spring, which may in turn stimulate the responses of the deep-brain photoreceptors responsible of photoperiodic time measurement in birds [26] and eventually lead to an earlier development of the reproductive system. Indeed, in female great tits (*Parus major*) a single long day can stimulate LH secretion and follicle growth [40]. However, erratic and variable levels of night-time illumination might be simply not enough to represent a constant long daylength that could prevent birds from breaking photorefractoriness. Although captive studies will still be able to provide unique insights into the physiological mechanisms underlying behavioural responses to light pollution, we suggest that future work should aim at investigating long-term consequences of light at night in the wild. If captive studies are to be done, then we suggest that animals should be given the possibility of hiding away from the light source, in order to better mimic real situations encountered in urban areas. Nevertheless, our data suggest that an uncontrolled increase in the amount of artificial light at night could pose serious risks for the reproductive capacity of avian species thriving in urban areas. Light at night has been already indicated as a public health issue by human studies [41,42] and possible remedies have been suggested [43,44]. Conversely, while effects of light pollution on wild animals are starting to be elucidated [1], little has been done to limit the impact of artificial lighting on ecosystems [45]. With the proportion of human population living in urban agglomerates projected to increase in the next 50 years [46], we suggest that a parallel increase in the amount of artificial light at night is not unlikely. It is therefore high time for scientists and policy-makers to start discussing ways to mitigate the ecological impacts of artificial light at night. 
